# Regularity detection under stress: Faster extraction of probability-based regularities

**DOI:** 10.1371/journal.pone.0253123

**Published:** 2021-06-15

**Authors:** Eszter Tóth-Fáber, Karolina Janacsek, Ágnes Szőllősi, Szabolcs Kéri, Dezso Nemeth

**Affiliations:** 1 Doctoral School of Psychology, ELTE Eötvös Loránd University, Budapest, Hungary; 2 Institute of Psychology, ELTE Eötvös Loránd University, Budapest, Hungary; 3 Institute of Cognitive Neuroscience and Psychology, Research Centre for Natural Sciences, Budapest, Hungary; 4 Centre of Thinking and Learning, Institute for Lifecourse Development, School of Human Sciences, Faculty of Education, Health and Human Sciences, University of Greenwich, London, United Kingdom; 5 Department of Cognitive Science, Budapest University of Technology and Economics, Budapest, Hungary; 6 National Institute of Psychiatry and Addictions, Nyírő Gyula Hospital, Budapest, Hungary; 7 Lyon Neuroscience Research Center (CRNL), INSERM U1028, CNRS UMR5292, Université de Lyon 1, Université de Lyon, Lyon, France; University of Utah, UNITED STATES

## Abstract

Acute stress can crucially influence learning and memory processes. One of the key processes underlying human learning and memory is the ability of our brain to rapidly detect and extract regularities from sensory input across time and space leading to effective predictive processing. Here, we aimed to get an in-depth look into the effect of stress on the acquisition of two aspects of regularity extraction. We examined whether and how stress affects the learning (1) of probability-based regularities and (2) of serial order-based regularities in the same experimental design, and (3) the explicit access to the acquired information. Considering that the acquisition of probability-based regularities is a relatively rapid process, we primarily focused on the early phase of the task. We induced stress with the Socially Evaluated Cold Pressor Test in 27 young adults, while 26 participants were enrolled in the control group. Salivary cortisol levels and subjective ratings of affective states showed successful stress induction. After the stress induction, we measured regularity extraction with the cued Alternating Serial Reaction Time task. We found that stress promoted the extraction of probability-based regularities measured by the learning performance in the early phase of the task and did not alter the learning of serial order-based regularities. Post-block reports showed weaker explicit access to the serial order-based regularities in the stress group. Our results can contribute to a process-level understanding on how stress alters learning and memory functions related to predictive processes.

## Introduction

Stressful situations are ubiquitous parts of our everyday life. Acute stress leads to changes in both cognitive and affective processes [1–4]. Previous studies have shown the crucial influence of stress on learning and memory functions [e.g., 5–7]. One of the key processes underlying human learning and memory is the ability of our brain to rapidly extract regularities from sensory input across time and space [8–11]. This knowledge is then used to update our predictions of future events [12, 13]. The aim of the present study is to determine how stress affects two aspects of such regularity extraction.

The available information in our environment is diverse, we can detect and extract several different kinds of regularities. Extraction of regularities can be considered as a form of statistical learning, which has been defined as the ability to pick up probabilities from the environment: people are exposed to structured input patterns over multiple times and acquire the regularities incidentally [11, 14, 15]. Humans are highly proficient in extracting transitional probabilities, i.e., in the learning of predictive relations between elements, even when these elements are nonadjacent [11, 16]. Transitional probabilities can range from zero (no predictability) to one (full predictability). A transitional probability of 1.0 means that an outcome can be predicted with 100% certainty, therefore, a transitional probability of 1.0 creates a deterministic serial order (or deterministic sequence) of events. We refer to this aspect of regularity extraction as learning of serial-order information. Transitional probabilities can be less than 1.0 as well, where higher transitional probabilities mean higher predictability. Prior studies have shown that humans learn to differentiate between more and less probable outcomes [e.g., 17, 18]; that is, they are highly sensitive to learning probability-based regularities where the transitional probabilities are less than 1.0.

As described above, from a theoretical perspective, both aspects of learning investigated in the current study can be viewed as a form of statistical learning, i.e., as learning of transitional probabilities. Nevertheless, a growing body of research shows that the learning of serial-order and probability-based regularities are distinguishable on both neural and behavioral levels: they develop differently at the level of event-related potentials [19, 20], and they show distinct neural oscillations during consolidation [21]. Crucially, on the behavioral level, participants acquire probability-based regularities rapidly and show consistent, stable performance thereafter [19, 21]. Simor et al. [21] explored the trajectory of learning probability-based regularities in detail. The results showed that picking up such regularities from the stimulus stream happens very quickly and it is not due to pre-existing tendencies. The results also indicated that additional training helps strengthen the acquired knowledge. In contrast, acquiring serial-order information seems to require more time, suggested by the gradual learning trajectory [19, 21]. Considering the extant evidence showing that the learning of these regularities is distinguishable [19–22], we will refer to them as learning of serial-order information (where second-order transitional probabilities are equal to 1.0) and learning of probability-based information (where second-order transitional probabilities are less than 1.0) in the rest of the paper. In the present study, we aimed to investigate how stress might affect two aspects of regularity extraction, namely, the learning of serial-order regularities and probability-based regularities.

The extraction of probability-based and serial-order regularities from the environment enables us to adapt to our surroundings and has been proposed to contribute to the acquisition of skills and habits [i.e., procedural memory, 17, 22]. Hence, both kinds of regularities are highly relevant in cognitive, motor and even social skills. Stress and stressful events are powerful modulators of learning. Several studies have investigated the influence of stress on different memory systems, such as declarative [e.g., 23, 24] and procedural memory [e.g., 7, 25], skill learning [e.g., 26–30] and habits [e.g., 31–33]. However, to the best of our knowledge, only a handful of studies investigated the effect of stress particularly on the acquisition of probability-based or serial order-based regularities [4, 34, 35]. In King’s [35] study, participants were exposed to probability-based regularities without being aware that learning was taking place. Stressed participants showed overall enhanced learning in accuracy. Regarding the acquisition of serial order-based regularities, Römer et al. [34] examined the effect of exogenous cortisol administration on learning deterministic second-order conditional sequences using a serial reaction time task. While both the cortisol and placebo groups showed significant learning, cortisol changed the time course of learning. The cortisol group showed delayed learning with decreased performance at the beginning of the task and equaled performance at the end of the task. In contrast, Dolfen et al. [4] did not find any effect of experimental stress exposure on learning deterministic sequences in a bimanual finger-tapping task. To sum up, only three studies examined whether stress influences the extraction of probability- or serial order-based regularities.

Altogether, the abovementioned studies do not allow us to draw strong conclusions about whether and how stress influences the acquisition of different types of regularities that are extracted from the environment. Thus, the aim of the present study was to test the effect of stress on two aspects of regularity extraction, namely the acquisition of probability-based and serial order-based information. As prior studies are scarce and inconclusive, we use an exploratory approach to investigate this question. As the extraction of probability-based information is a rapid process [9, 11, 19, 21], if stress affects this aspect of learning, we expect it to occur at the beginning of the task, irrespective of whether stress diminishes or enhances the acquisition. In the case of acquiring serial-order information, as it typically shows a gradual trajectory [19, 21], if stress affects this aspect of learning, we expect the alterations to occur in the later stages of the task.

## Materials and methods

### Participants

Sixty-five healthy undergraduates enrolled in our study. Two participants’ baseline salivary cortisol levels were more than three standard deviations away from the mean of the sample. Therefore, these two participants were considered as outliers, and their data were not included in the analyses. Three participants were also excluded from the analyses because they did not provide sufficient saliva for cortisol analysis. Additionally, as following instructions in the cued ASRT task (for details, see Regularity extraction task section) is crucial, seven participants were excluded as they did not meet this requirement (for details, see Statistical analysis section; for analysis on the sample containing these participants, see S1 File).

The final sample consisted of 53 participants (15 men, 38 women; M_age_ = 20.9 years, SD = 1.7). Participants were randomly assigned to either the control (7 men, 19 women) or the stress (8 men, 19 women) group. 10 women in the stress group and 7 women in the control group took oral contraceptives regularly. All participants had normal or corrected-to-normal vision, none of them reported a history of any psychiatric, neurological or any other chronic medical problems. They performed in the normal range on standard neuropsychological tests (Counting Span task: M_stress_ = 3.87, M_control_ = 3.73; Digit Span task: M_stress_ = 7.03, M_control_ = 7.23), with no difference between the stress and control groups (all *p*s > .537, Cohen’s *d* < 0.176). Prior to our study, the sample size was determined using ‘best practices’. Thus, we took into account the sample sizes in previous studies that employed either the uncued or the cued ASRT task in an adult population with similar study design as ours [i.e., comparing the learning of two or more groups, 21, 22, 36–41]. The sample sizes varied between 14 and 63 per group, with an average of 27.33 (*SD* = 11.82). Based on this, assessing 25–35 participants per group seemed reasonable. Moreover, we fulfilled every requirement stated in the study of Simmons et al. [42].

Participants were recruited from a university course in exchange for course credit. All participants provided written informed consent before enrolment. The study was approved by the research ethics committee of the Eötvös Loránd University in Hungary and was conducted in accordance with the Declaration of Helsinki.

### Experimental design

To exclude the effect of critical factors on salivary cortisol levels, participants were asked not to eat, drink (except for water), smoke, and make physical exercise two hours prior to the experiment [see 43–46]. To avoid interference with the cortisol circadian cycle [see 47], assessments took place between 1 and 7 p.m.

**Fig 1:**
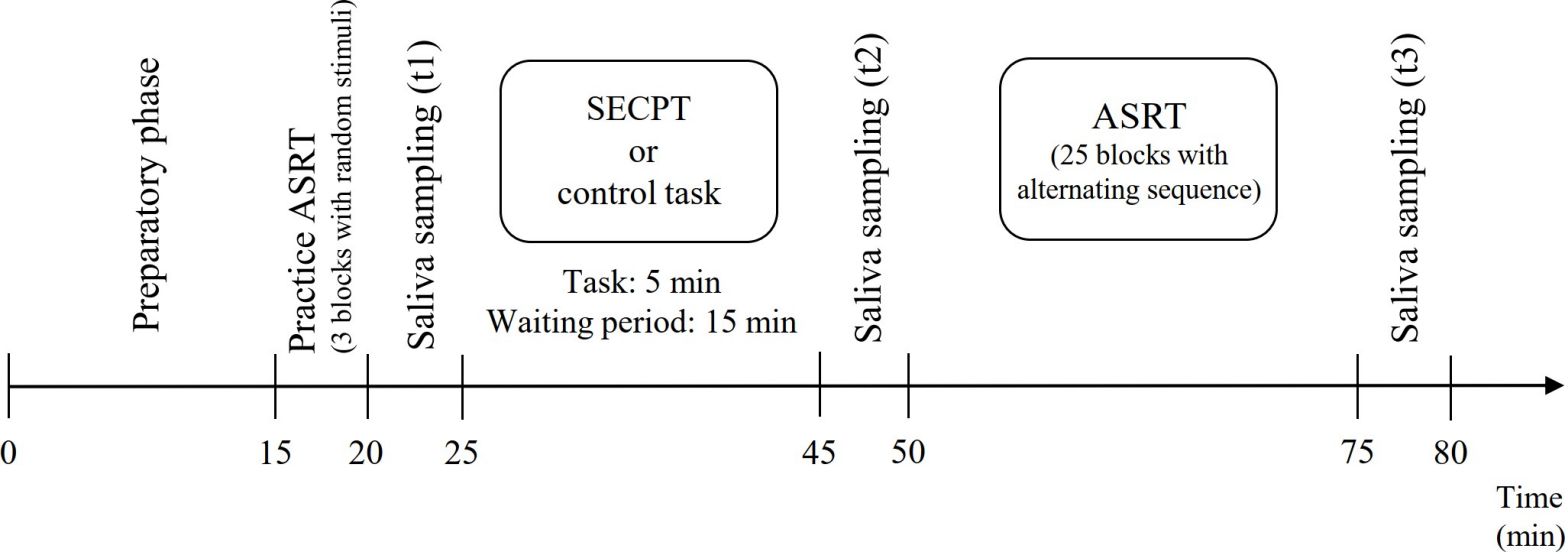
Experimental procedure. Cortisol levels were assessed from saliva at three time points: immediately before the stress induction or control task (t1), 15 minutes after the stress induction or control task (t2), and immediately after the ASRT task, ca. 45 minutes after the stress induction or control task (t3). ASRT: Alternating Serial Reaction Time task; SECPT: Socially Evaluated Cold Pressor Test.

Each experimental session started with a 15-min preparatory phase while participants remained in a preparatory room for 15 minutes to minimize the effect of potentially stress-inducing factors [such as new environment; see 48]. Then, participants completed a practice session (3 blocks, ca. 3 minutes) of the regularity extraction task. Note that in the practice session stimulus appeared randomly, hence, it could not influence the learning of probability-based and serial order-based regularities. To measure cortisol levels, saliva samples were collected at different time points during the experimental session (see Fig 1). The first saliva sampling was conducted immediately after the practice session of the regularity extraction task. This baseline saliva sampling was followed by the stress induction or control task (see the details below). To assess subjective stress levels, immediately after the stress induction or the control task, participants were required to rate how stressful (0 = not at all, 100 = very stressful), painful (0 = not at all, 100 = very painful), and unpleasant (0 = not at all, 100 = very unpleasant) the stress induction or the control task was. The second saliva sample was collected 15 minutes after stressor offset/the end of the control task, followed by the regularity extraction task. The third saliva sample was collected immediately after the regularity extraction task (approximately 40 minutes after stressor offset/the end of the control task). Samples were collected using Eppendorf Safe-Lock Tubes (1.5 ml), were kept at -20°C between the experimental session and the analysis. Saliva samples were assayed using a Salimetrics kit at the National Institute of Psychiatry and Addictions, Budapest, Hungary. The inter-assay coefficient of variability was 8%, and the intra-assay coefficient of variability was 5%.

### Stress induction task and control condition

Individuals in the stress group participated in the Socially Evaluated Cold Pressor Test [49]. This task was developed to induce moderate stress in laboratory settings using both physical and psychosocial stressors. Participants were instructed to put their hand into cold water (0–3°C) for three minutes while a female experimenter observed their behavior. The observer did not interact with the participants, just passively monitored their behavior. Participants were also told that a video recording would be made to later analyze their non-verbal behavior. They were informed that they could interrupt the task if it would be too painful or too uncomfortable.

There were no stress inducing factors (i.e., cold water, observer, and video recorder) in the control condition. Control participants were required to put their hand into warm water (35–37°C) for three minutes. A female experimenter stayed in the experimental room (just as in the stress condition), however, this time she did not observe participants’ behavior.

### Regularity extraction task

Regularity extraction was measured by the cued version of the Alternating Serial Reaction Time (ASRT) task [21, 22, 50]. In this task, a target stimulus (either a dog’s head or a penguin) appeared in one of the four possible locations, which were horizontally arranged and equally spaced empty circles. Participants were asked to press the corresponding key on the keyboard (Z, C, B, or M) as accurately and as fast as they could, using their index- and middle-fingers of both hands. The stimulus remained on the screen until the participant’s response, then, after a 120 ms delay, the next target appeared (note that in this task, one stimulus corresponds to one trial).

Participants started the experimental session with a practice session of the ASRT task consisting of 3 blocks with 85 trials each. Importantly, in the practice session, stimulus appeared randomly, that is, without any predetermined sequence. In the task, however, the presentation of the stimuli followed an 8-element alternating sequence where pattern and random trials alternated with each other (e.g., 1-r-2-r-4-r-3-r, where numbers represent the predetermined locations from left to right and ‘r’ indicates a randomly selected location). The pattern and random trials were marked differently, pattern trials indicated by the dog’s head, and random trials represented by the penguins. The alternation of pattern and random trials creates six distinguishable sequence permutations: 1-r-2-r-3-r-4-r, 1-r-2-r-4-r-3-r, 1-r-3-r-2-r-4-r, 1-r-3-r-4-r-2-r, 1-r-4-r-2-r-3-r, and 1-r-4-r-3-r-2-r. One of these permutations was selected for each participant and it was counterbalanced between groups and also across participants in each group.

Participants were told that the appearance of the dogs always followed a predetermined sequence, while penguins always appeared on a randomly chosen location. They were instructed to find the pattern defined by the dog’s appearance to improve their performance. They had no information about the length of the predetermined sequence. Marking the pattern and random trials with different visual stimuli and instructing participants to find the pattern was employed to speed up learning. The differentiation of probability-based and serial-order regularities is possible in the uncued, implicit version of the ASRT task as well (where pattern and random trials are marked with the same visual stimuli), however, serial-order knowledge requires extensive practice to develop [17]. Visual distinctions between pattern and random trials together with the instruction to find the pattern help participants to acquire the serial-order knowledge in a faster manner, enabling us to measure the learning of probability-based and serial-order regularities in the same time frame (i.e., within one learning session).

The task contained 25 blocks and each block consisted of 85 trials. The first five trials of each block were random trials for practice, then the unique 8-element predetermined sequence was presented 10 times. After each block, participants were instructed to type the order of the dog’s head using the corresponding keys to measure awareness of the sequence structure. This post-block sequence report lasted until the participants gave 12 consecutive responses, which are ideally the 4-element sequence three times. For these post-block sequence reports, we calculated how many responses out of the total 12 were correct after each block, expressed in terms of percentages. The mean of these block-level values was calculated in each epoch (i.e., 5 blocks) for each participant and used as measures of their explicit sequence knowledge (that is, knowledge on the deterministic serial order of pattern stimuli) acquired during the task.

The alternating sequence in the ASRT task makes some runs of three successive trials–referred to as triplets–more probable than others. For example, if the sequence is 1-r-2-r-4-r-3-r, triplets such as 1-X-2, 2-X-4, 4-X-3, 3-X-1 (where X indicates the middle element of the triplet) occur with a higher probability since their last trial can be either predetermined or random. However, 3-X-2 or 4-X-2 occur with a lower probability as the third trial could only be random. The more probable triplet types are hereinafter referred to as high-probability triplets and the latter types as low-probability triplets [17, 22]. Since high-probability triplets can end with a pattern trial due to the predetermined sequence or with a random trial by chance, we can differentiate between pattern high-probability and random high-probability triplets. Low-probability triplets can only end with a random trial, as pattern trials are always at high probability. Each trial is categorized as the last element of a triplet in a moving window manner, meaning that a given trial is categorized as the first element of a given triplet, then as the second element of the following triplet, and so on. Each trial was categorized as the third element of either a high-probability or a low-probability triplet and also either as pattern or random elements. There are 64 unique triplets in the task, including all pattern-ending (50%) and random-ending (50%) triplets. Sixteen of these unique triplets are of high-probability and 48 triplets are of low-probability. As high-probability triplets can occur as pattern-ending triplets (50% of all trials) and by ¼ chance as random-ending triplets (12.5% of all trials), these triplets constitute 62.5% of all trials (Fig 1C). Low-probability triplets constitute 37.5% of all trials. On the level of unique triplets, high-probability triplets are five times more probable than low-probability triplets (4% [62.5% / 16] vs. 0.8% [37.5% / 48]). In sum, three trial types can be differentiated: (1) trials that belong to the predetermined sequence and are the final element of a high-probability triplet labeled as *pattern trials*, (2) trials that occur randomly and also are the final element of a high-probability triplet called *random high trials*, and (3) random elements that appear as the last element of a low-probability triplet called *random low trials*.

**Fig 2:**
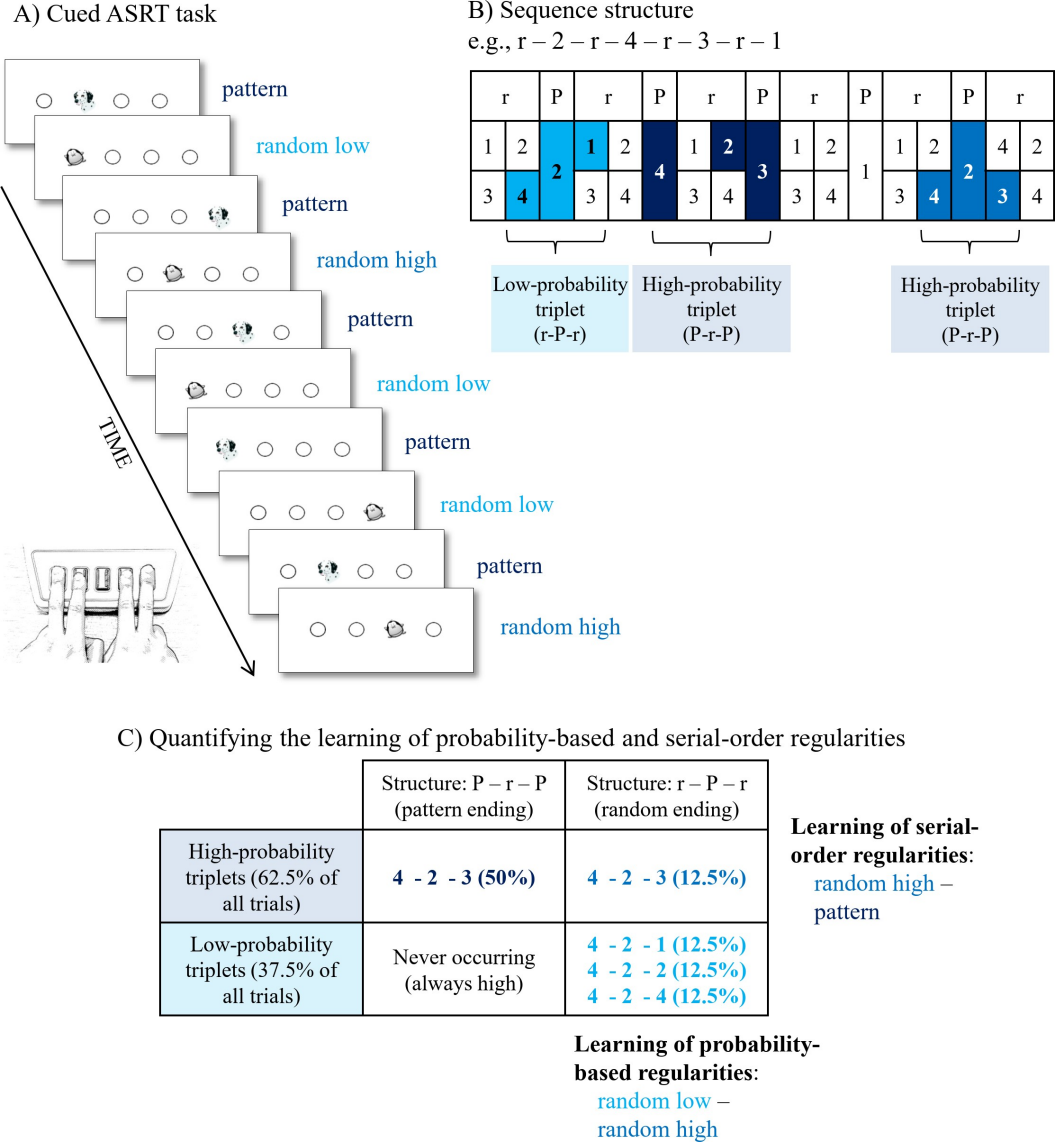
The cued Alternating Serial Reaction Time (ASRT) task. (A) Pattern and random trials were presented in an alternating fashion in the ASRT task. Pattern trials were indicated by a picture of a dog and random trials were indicated by a picture of a penguin. (B) An example of the alternating sequence structure. Here, numbers indicate pattern trials and ‘r’ indicates a randomly selected location. The alternating sequence makes some runs of three consecutive trials (called triplets) more probable than others, termed high- and low-probability triplets, respectively. Among high-probability triplets, we determined pattern high-probability triplets (dark blue shading in panel B and dark blue font in panel C) and random high-probability triplets (blue shading in panel B and blue font in panel C). Low-probability triplets can occur only in random positions (light blue shading in panel B and light blue font in panel C). (C) The underlying learning processes in the task. Learning of serial-order regularities is quantified by contrasting the RTs on the pattern and random high trials. Learning of probability-based regularities is quantified by contrasting the RTs on the random high and random low trials. Adapted from Németh et al. [22].

Based on these trial types, we can differentiate between two types of regularity extraction: learning of probability-based and serial-order regularities [Fig 2C; 17, 19, 21, 22]. Learning of probability-based information is measured by the difference in reaction times (RTs) between random high and random low trials. These trials share the same sequence structure as they are both random trials but differ in probabilistic properties as they correspond either to the final trial of a high-probability or a low-probability triplet. Greater learning of probability-based regularities is indicated by faster RTs on random high than on random low trials. Learning of serial-order regularities is measured by the difference in RTs between pattern and random high trials. These trials share the same probabilistic properties as they are both the last element of a high-probability triplet, but differ in sequence properties as pattern trials are part of the predetermined sequence, while random high trials appear on a random location. Greater learning of serial-order regularities is defined as faster RTs on pattern than on random high trials.

### Statistical analysis

Statistical analyses were carried out by SPSS version 25.0 software (SPSS, IBM). As participants were instructed to find the alternating sequence and report that during the task, performance on the post-block sequence report (calculated as the average percentage of correctly reporting the deterministic serial order of the pattern stimuli after each of the ASRT task blocks) could show whether participants adequately followed the instructions of the task. In the present study, seven participants exhibited below 50% performance, suggesting that they did not follow the instructions properly and were therefore excluded from the analyses (see also Participants section; for analysis on the sample containing these participants, see S1 File).

Statistical analysis of the effectiveness of stress induction was the same as in prior studies [e.g., 51, 52]. We conducted a mixed design ANOVA on cortisol levels as dependent variable, with TIME (t1, t2, and t3) as a within-subject factor, where t1 = immediately before stress/control procedure, t2 = 15 minutes after stress/control procedure, t3 = 45 minutes after stress/control procedure, and with STRESS EXPOSURE (stress or control) as a between-subject factor. The effectiveness of stress induction was also quantified with subjective variables. Immediately after the stress induction or control task, participants were asked to rate how stressful, painful, and unpleasant the stress induction or the control task was. We compared these subjective ratings of affective state between the stress and control groups with independent sample t-tests. Moreover, as oral contraceptive use and menstrual cycle could affect cortisol response [53], we checked whether these factors influenced cortisol response in women in the stress group. Using independent samples t-test, we compared cortisol response (i.e., baseline cortisol level extracted from the cortisol level measured 15 minutes after the offset of stressor) of those women who use oral contraceptive (*n* = 10) and those who do not (*n* = 9). As for menstrual cycle, during testing, we asked women the following questions about their cycle: (1) when the first and last day of their last menstruation was and (2) what the average length of their menstrual cycle is. Due to the lack of hormonal testing, we cannot reliably determine all phases of the menstrual cycle. Hence, we followed a conservative approach: women who were on the 1–12 days of their cycle on the day of testing were assigned to the follicular phase group (*n* = 6), whereas women who were equal to or more than 17 days away from the start of their last menstruation, were placed in the luteal phase group (*n* = 7). Then we used independent samples t-test to compare cortisol response in these two subgroups. Participants who were around mid-cycle and therefore possibly ovulating were excluded from this analysis.

To assess participants’ average speed prior to stress induction or control task, and to exclude the possibility of pre-existing group differences influencing the effect of stress on learning, we conducted a mixed design ANOVA on the practice session of the ASRT task. This session consisted of three blocks and the stimuli were presented randomly, therefore average RTs could be assessed. The mixed design ANOVA involved GROUP (stress vs control) as a between-subject factor and BLOCK (1–3) as a within-subject factor.

Statistical analysis of the ASRT task was based on previous studies [19, 21, 22]. Two types of low-probability triplets, namely repetitions (e.g. 111, 222) and trills (e.g. 121, 242) were excluded from the analysis, since participants often show pre-existing tendencies to these trials [50, 54]. Following prior studies [e.g., 19, 21, 22, 36, 55], the ca. 1-minute-long blocks were organized into larger segments labeled as epochs. Each epoch consists of five blocks, thus, we analyzed five epochs of the ASRT task.

As described in the Regularity extraction task section, each trial was determined as the last element of a *pattern high*, *random high*, or *random low* triplet. The median of RT data for correct responses was calculated for each participant in each epoch, separately for the three trial types. As presented above, two aspects of regularity extraction, the learning of probability-based information and serial-order information can be assessed by the ASRT task [22; for further details, see Introduction and the task description above]. To examine the two aspects, RT data were analyzed in a mixed design ANOVA. At first, as learning of probability-based regularities is a rapid process [19, 21], we focused on the early phase of the task. We used a mixed design ANOVA with PROBABILITY (random high vs random low trials) as a within-subject factor and GROUP (stress vs control) as a between-subject factor on the RT data showed in the first epoch. Similarly, at first, learning of serial-order regularities was compared only in the last epoch of the task as this learning process shows a gradual trajectory [19, 21], and therefore, the effect of stress may emerge in this later phase of learning. Hence, first, we used a mixed design ANOVA with ORDER (pattern vs random high trials) as a within-subject factor and GROUP (stress vs control) as a between-subject factor on the RT data showed in the last epoch.

Next, to investigate learning in the learning phase (i.e., on the whole task), learning of probability-based regularities was quantified with a mixed design ANOVA with PROBABILITY (random high vs random low trials) and EPOCH (1–5) as within-subject factors and GROUP (stress vs control) as a between-subject factor. Similarly, learning of serial-order regularities was quantified with a mixed design ANOVA with ORDER (pattern vs random high trials) and EPOCH (1–5) as within-subject factors and GROUP (stress vs control) as a between-subject factor. Pairwise comparisons were performed using LSD (Least Significant Difference) to control for type 1 error. The Greenhouse-Geisser epsilon correction was used when necessary. Original *df* values and corrected *p* values (if applicable) are reported together with partial eta-squared (*η*^*2*^_*p*_) as a measure of effect size.

The analysis on the learning of probability-based information showed that, on average, the stress group showed faster RTs compared to the control group. Therefore, we transformed the raw RT data into standardized RTs (for a detailed description, see Results). In conjunction with the analyses on raw RT data, we also present identical analysis on standardized RT data to ensure that potential group differences are not masked by the group differences in average RTs.

## Results

### The effectiveness of stress induction

**Fig 3:**
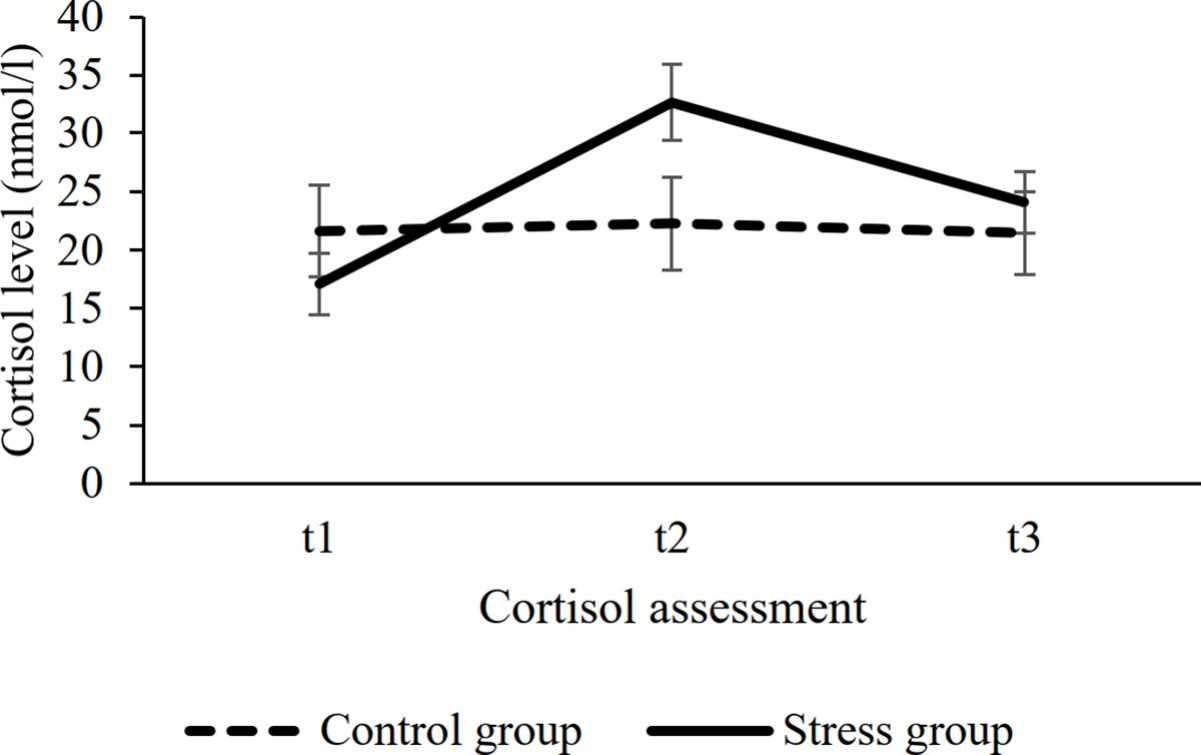
The effectiveness of stress induction. Salivary cortisol levels were assessed immediately before (t1), 15 minutes after (t2), and 45 minutes after (t3) the stress induction or control task. Error bars denote the standard error of the mean (SEM).

We used both objective (cortisol levels) and subjective (questionnaire-based rating) measurements to test the effectiveness of stress induction. For the *objective measurement*, the ANOVA confirmed the effectiveness of the stress induction. We found a significant TIME × STRESS EXPOSURE interaction (*F*(2, 102) = 29.75, *p* < .001, *η*^*2*^_*p*_ *=* .368). The post hoc analysis revealed that cortisol levels in the stress and control groups differed 15 minutes after the stress/control procedure (t2; *M*_stress_ = 32.62 and *M*_control_ = 22.26, respectively, *p* = .048), while immediately before the stress/control procedure (t1) and immediately after the ASRT task (t3) the two groups’ cortisol levels did not differ significantly (both *p*s > .34, see Fig 3; for a figure with individual data points, see S4 Fig in S1 File). For the *subjective ratings* of affective state after stress induction or control task, the t-tests showed significantly higher subjective stress, pain, and unpleasantness levels in the stress group compared to the control group (see Table 1).

**Table 1:** Descriptive data of the participants’ subjective stress measurements.

	Group					
	Stress		Control			
	(*n* = 27)		(*n* = 26)			
	*M*	*SD*	*M*	*SD*	*t*	*p*
Subjective ratings of stress	29.88	26.14	8.34	12.43	-3.85	< .001**
Subjective ratings of unpleasantness	56.29	28.78	11.30	14.29	-7.24	< .001**
Subjective ratings of painfulness	58.14	25.56	1.34	5.92	-11.23	< .001**

*Note*. Immediately after the stress induction or control task, participants rated how stressful, unpleasant, and painful (where 0 = not at all, 100 = very) the stress induction or control task was.

**p < .001.

For the effects of oral contraceptive use on cortisol response (i.e., baseline cortisol level extracted from the cortisol level measured 15 minutes after the offset of stressor) in the stress group, the independent samples t-test showed that cortisol response of women who use oral contraceptives and of those who do not use them did not differ significantly (*t*(17) = 0.35, *p* = .73). Cortisol response also did not differ significantly in the follicular and luteal phase groups (*t*(11) = -0.46, *p* = .65).

### Average RTs in the practice session

To compare the two groups’ average speed in the practice session of the ASRT task, a mixed design ANOVA was conducted. The ANOVA revealed that participants became faster as the task progressed (main effect of BLOCK, *F*(2, 102) = 59.11, *p* < .001, *η*^*2*^_*p*_ *=* .54). Importantly, no group differences were found either in average RTs (main effect of GROUP, *F*(1, 51) = 0.17, *p* = .68) or in the speed-up during the practice blocks (GROUP × BLOCK interaction, *F*(2, 102) = 0.29, *p* = .72).

### The effect of stress induction on the learning of probability-based regularities

As learning of probability-based regularities is a rapid process [19, 21], we focused on the beginning of the task. Therefore, we first examined learning of probability-based regularities only in the first epoch of the learning phase using a mixed-design ANOVA (see Statistical analysis section) between the stress and control groups. Overall, there was no difference in average RTs between the two groups (main effect of GROUP, *F*(1, 51) = 2.67, *p* = .11). Participants responded faster to random high than random low trials (shown by the significant main effect of PROBABILITY, *F*(1, 51) = 32.34, *p* < .001, *η*^*2*^_*p*_ *=* .39), indicating significant learning of probability-based information. Importantly, the stress group showed greater learning of probability-based regularities than the control group (indicated by the significant PROBABILITY × GROUP interaction, *F*(1, 51) = 4.07, *p* = .05, *η*^*2*^_*p*_ *=* .07; stress group: *M*_*random high*_ = 373.07 ms, *M*_*random low*_ = 390.59 ms, learning score: *M* = 17.52 ms; control group: *M*_*random high*_ = 401.69 ms, *M*_*random low*_ = 410.04 ms, learning score: *M* = 8.35 ms).

To explore the trajectory of learning of probability-based information in the stress and control groups, we conducted a mixed-design ANOVA on the RTs over the entire learning phase (see Statistical analysis section). This analysis revealed significant differences in average RTs between the two groups with the stress group showing, on average, faster RTs (*M* = 371.19 ms) than the control group (*M* = 391.46 ms) (shown by the significant main effect of GROUP, *F*(1, 51) = 3.98, *p* = .05, *η*^*2*^_*p*_ *=* .07). Participants became faster with practice, irrespective of trial type (shown by the significant main effect of EPOCH, *F*(4, 204) = 4.30, *p* = .03, *η*^*2*^_*p*_ *=* .08). Importantly, RTs were faster on random high trials than on random low trials (indicated by the significant main effect of PROBABILITY, *F*(1, 51) = 139.46, *p* < .001, *η*^*2*^_*p*_ *=* .73), revealing significant learning of probability-based information. None of the interactions reached significance (all *p*s > .12, see Table 2).

**Table 2:** Summary of results from ANOVAs performed on raw RT and standard RT data considering learning of probability-based regularities.

	raw RT			standard RT		
	*F*	*p*	*η*^*2*^_*p*_	*F*	*p*	*η*^*2*^_*p*_
GROUP	3.98	.05*	.07	< 0.001	.99	< .001
PROBABILITY	139.46	< .001**	.73	142.05	< .001**	.74
EPOCH	4.30	.03*	.08	3.31	.05*	.06
GROUP × PROBABILITY	1.68	.20	.03	3.45	.07*+*	.06
GROUP × EPOCH	0.70	.46	.01	0.81	.42	.02
PROBABILITY × EPOCH	1.91	.12	.04	1.70	.16	.03
GROUP × PROBABILITY × EPOCH	1.57	.19	.03	1.38	.24	.03

Note.

**p < .01

*p < .05, +*p* < .10.

The group difference in average RTs revealed by this analysis could affect potential group differences in learning of probability-based information as well, for example, by masking differences in learning as the stress group had less room to improve on the task due to generally faster RTs. To control for the differences in average RTs, we transformed the data in the following way. We divided each participants’ raw RT values of each trial type and each epoch by their own average performance of the first epoch of the task [for a similar approach, see 56, 57], then we multiplied these values by 100 for easier presentation and interpretability. Participants’ performance was around 100 at the beginning of the task and changed as the task progressed. On these standardized RT data, we conducted identical analyses as on the raw RT data.

Firstly, we compared learning in the first epoch using a mixed-design ANOVA. The results were identical to the ones on raw RT data. Overall, the groups showed comparable average RTs (main effect of GROUP, *F*(1, 51) = 1.13, *p* = .29). Participants responded faster on random high than on random low trials (shown by the main effect of PROBABILITY, *F*(1, 51) = 32.59, *p* < .001, *η*^*2*^_*p*_ *=* .40), indicating significant learning of probability-based information. Moreover, the stress group showed greater learning in the first epoch than the control group (shown by the significant PROBABILITY × GROUP interaction, *F*(1, 51) = 4.46, *p* = .04, *η*^*2*^_*p*_ *=* .08).

**Fig 4:**
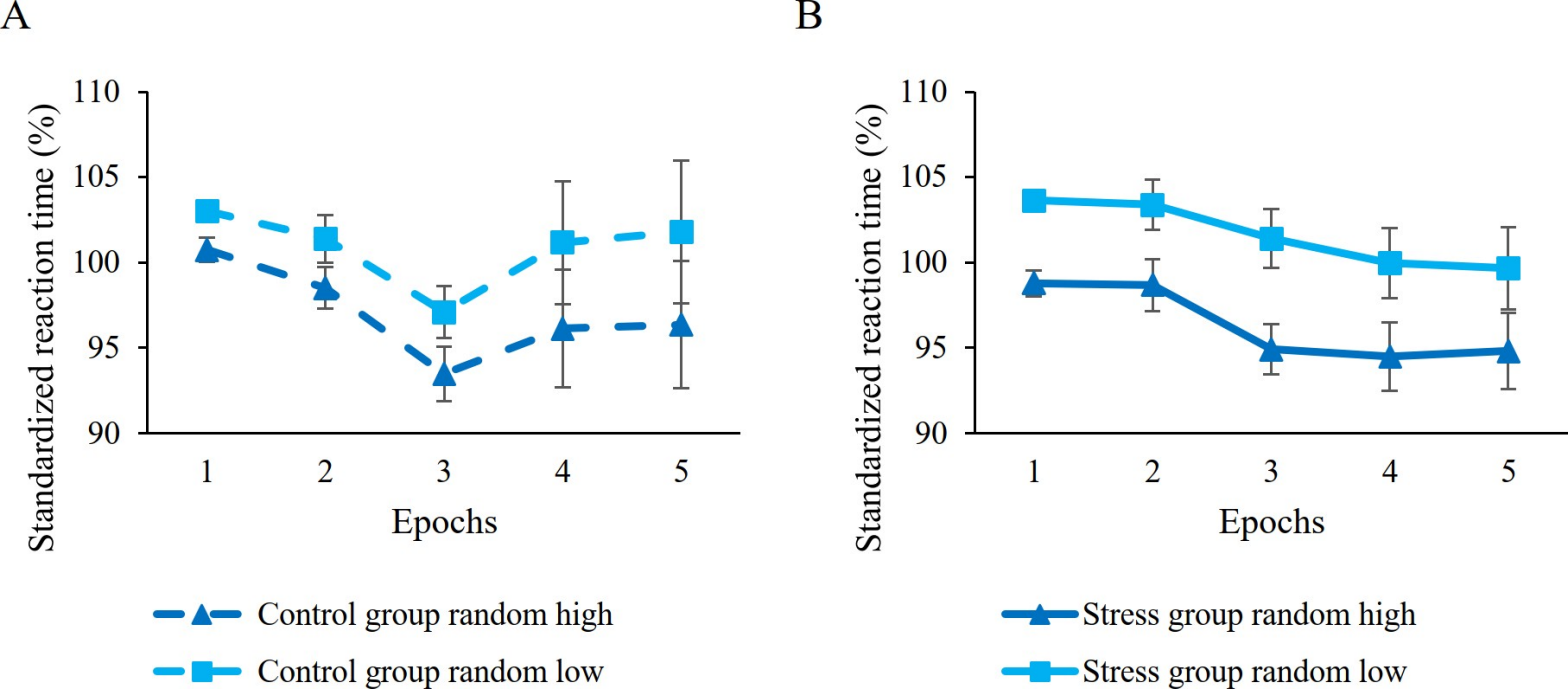
Learning of probability-based regularities in (A) control and (B) the stress groups. Dashed lines represent the control group, continuous lines represent the stress group. Blue lines with triangle symbols indicate standardized reaction times on the random high trials, light blue lines with square symbols indicate standardized reaction times on the random low trials. Learning of probability-based regularities is quantified by the distance between dashed and continuous lines, greater distance represents better learning. Error bars denote the SEM.

We also tested the trajectory of learning on the whole task using the standardized RT data. The previously seen group difference in average RTs has been controlled for by the standardization of the data (confirmed by the non-significant main effect of GROUP, *F*(1, 51) < .001, *p* = .98). Identically to the results on raw RT data, analysis showed that participants became faster as the task progressed, irrespective of trial types (main effect of EPOCH, *F*(4, 204) = 3.31, *p* = .05, *η*^*2*^_*p*_ *=* .06) and showed significant learning of probability-based regularities (main effect of PROBABILITY, *F*(1, 51) = 142.05, *p* < .001, *η*^*2*^_*p*_ *=* .74). Crucially, this analysis revealed that, overall, the stress group showed marginally greater learning of probability-based information than the control group (as indicated by the marginally significant GROUP × PROBABILITY interaction, *F*(1, 51) = 3.45, *p* = .07, *η*^*2*^_*p*_ *=* .06; stress group: *M*_*random high*_ = 96.34%, *M*_*random low*_ = 101.61%, standardized learning score: *M* = 5.27%; control group: *M*_*random high*_ = 97.04%, *M*_*random low*_ = 100.89%, standardized learning score: *M* = 3.85%, see Fig 4; for a figure with individual data points, see S5 Fig in S1 File). For further details on other interactions, see Table 2.

### The effect of stress induction on learning of serial-order regularities

As learning of serial-order regularities is a gradual process [19, 21], at first, we investigated learning between the groups in the last epoch of the learning phase using a mixed-design ANOVA (see Statistical analysis section). Overall, participants showed similar average reaction times (main effect of GROUP, *F*(1, 51) = 1.21, *p* = .28). Participants responded faster to pattern than to random high trials (main effect of ORDER, *F*(1, 51) = 9.54, *p* = .003, *η*^*2*^_*p*_ *=* .16), indicating significant learning of serial-order regularities. The analysis showed comparable learning between the groups (shown by the non-significant ORDER × GROUP interaction, *F*(1, 51) = 0.76, *p* = .39).

To examine the trajectory of learning of serial-order regularities on the whole task, we also used mixed-design ANOVA on the RT (see Statistical analysis section). Average RTs did not differ between the groups (main effect of GROUP, *F*(1, 51) = 2.68, *p* = .11). With practice, participants showed faster RTs on both trials (shown by the significant main effect of EPOCH, *F*(4, 204) = 35.35, *p* < .001, *η*^*2*^_*p*_ *=* .409). Moreover, participants showed faster RTs on pattern trials compared to random high trials (shown by the main effect of ORDER, *F*(1, 51) = 14.48, *p* < .001, *η*^*2*^_*p*_ *=* .22), indicating significant learning of serial-order information. The RT differences between pattern and random high trials gradually increased with practice (shown by the significant ORDER × EPOCH interaction, *F*(4, 204) = 4.46, *p* = .03, *η*^*2*^_*p*_ *=* .08). Other interactions did not reach significance (all *p*s > .37, see Table 3).

**Table 3:** Summary of results from ANOVAs performed on raw RT and standard RT data considering learning of serial-order regularities.

	raw RT			standard RT		
	*F*	*p*	*η*^*2*^_*p*_	*F*	*p*	*η*^*2*^_*p*_
GROUP	2.68	.11	.05	0.08	.78	.002
ORDER	14.48	< .001**	.22	14.38	< .001**	.22
EPOCH	35.35	< .001**	.41	38.36	< .001**	.43
GROUP × ORDER	0.55	.46	.01	0.35	.57	.01
GROUP × EPOCH	1.01	.37	.02	0.84	.46	.02
ORDER × EPOCH	4.46	.03*	.08	4.75	.02*	.09
GROUP × ORDER × EPOCH	0.47	.55	.01	0.43	.57	.01

Note.

**p < .01

*p < .05.

Although, considering learning of serial-order regularities, the analysis involving raw RT data did not show differences in average RTs between the groups, for sake of completeness, we conducted identical analyses on standardized RT data as well. Firstly, we compared learning in the last epoch between the groups using mixed-design ANOVA. Similarly to the results on raw RT data, there was no difference in average RTs between the groups (main effect of GROUP, *F*(1, 51) = 0.57, *p* = .46) and participants showed faster RTs on pattern trials compared to random high trials (main effect of ORDER, *F*(1, 51) = 9.71, *p* = .003, *η*^*2*^_*p*_ *=* .16), suggesting significant learning of serial-order information. The analyses did not reveal any group differences in learning (shown by the non-significant ORDER × GROUP interaction, *F*(1, 51) = 0.61, *p* = .44).

**Fig 5:**
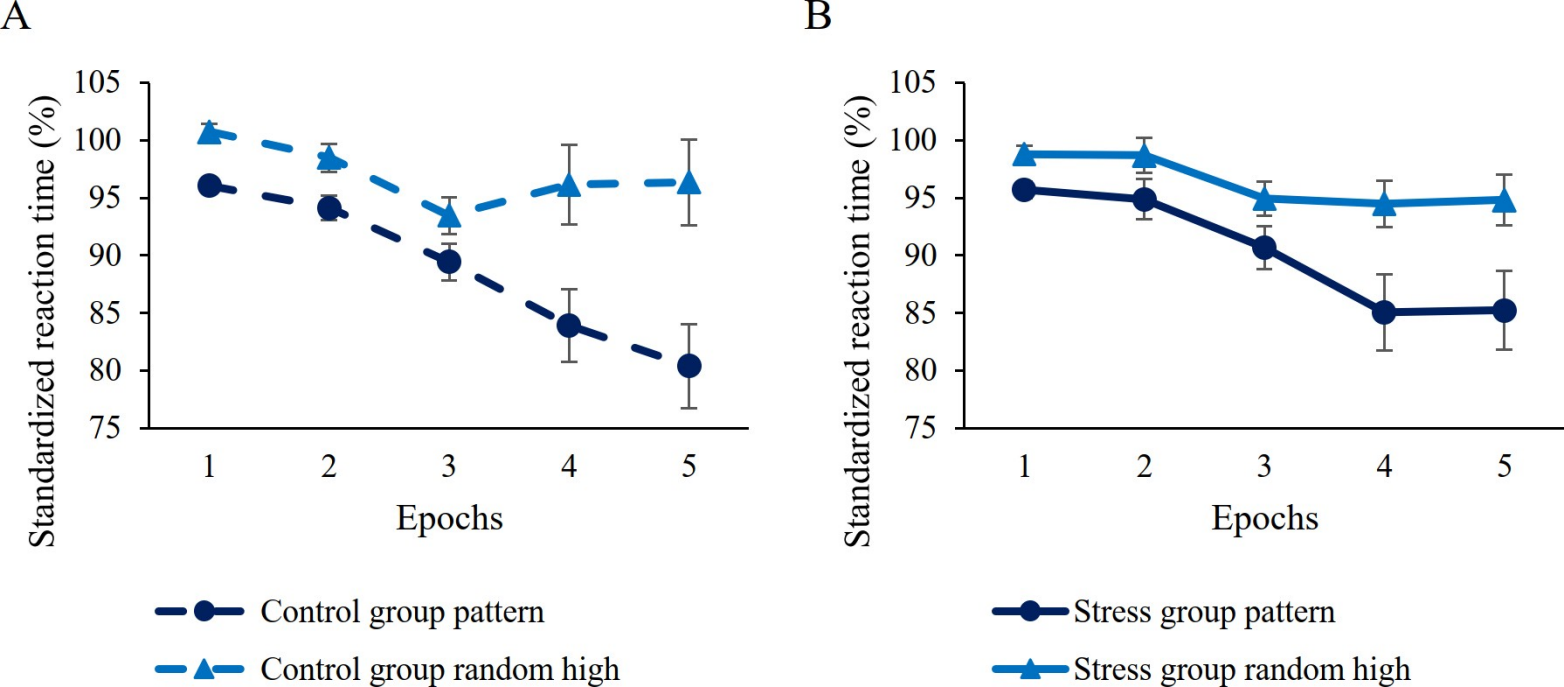
Learning of serial-order regularities in (A) control and (B) the stress groups. Dashed lines represent the control group, continuous lines represent the stress group. Dark blue lines with circle symbols indicate standardized reaction times on pattern trials, blue lines with triangle symbols indicate standardized reaction times on random high trials. Learning of serial-order regularities is quantified by the distance between dashed and continuous lines, greater distance represents better learning. Error bars denote the SEM.

To compare learning of serial-order regularities between the groups on the whole task as well, we used mixed-design ANOVA involving all five epochs of the standard RT data. The results were identical to the ones on raw RT data, overall showing gradual learning of serial-order regularities (indicated by the significant ORDER × EPOCH interaction, *F*(4, 204) = 4.75, *p* = .02, *η*^*2*^_*p*_ *=* .09; see Fig 5; for a figure with individual data points, see S6 Fig in S1 File), but not revealing any group differences (main effect of GROUP and any interaction involving GROUP were not significant, all *p*s > .46, see Table 3).

Performance on the post-block sequence reports, reflecting explicit knowledge of the deterministic serial order of the pattern stimuli, was also compared between the groups. To investigate the change in this knowledge during the task, we conducted a mixed-design ANOVA with GROUP (stress vs control) as a between-subjects factor and EPOCH (1–5) as a within-subject factor. Averaged over the epochs, the stress group showed lower explicit knowledge than the control group (main effect of GROUP, *F*(1, 51) = 4.32, *p* = .04, *η*^*2*^_*p*_ *=* .08, *M*_stress_ = 92.4%, *M*_control_ = 96.2%). This knowledge increased as the task progressed in both groups (*F*(4, 204) = 9.24, *p* < .001, *η*^*2*^_*p*_ *=* .15), however, the time course of this increase differed in the groups (indicated by the significant GROUP × EPOCH interaction, *F*(4, 204) = 5.05, *p* = .005, *η*^*2*^_*p*_ *=* .09). Pairwise comparisons showed that the control group had comparable explicit knowledge of the deterministic serial order of pattern stimuli in all epochs, which varied between 94.3% and 97.7% (all *p*s < .29). In contrast, the stress group showed significantly lower explicit knowledge in the first epoch (*M* = 80%) compared to the remaining epochs (*M* > 91.8%, all *p*s < .001). Then, they showed mostly comparable explicit knowledge of the serial order of pattern stimuli in Epoch 2–5, which varied between 91.8% and 98.4% (all *p*s > .09, except for the pairwise comparison between Epoch 2 and Epoch 5, where Epoch 2 < Epoch 5, *p* = .003).

## Discussion

To date, only three studies focused on how stress alters the detection and extraction of different types of regularities from the environment [4, 34, 35]. In the present study, we aimed to get a better understanding of how stress influences the simultaneous acquisition of probability-based and serial-order regularities. Our results, supported by analyses both in the main text and in the S1 File, showed that stress affected the extraction of probability-based regularities at the beginning of the ASRT task, while learning of serial-order information was unaffected by the stress induction. Moreover, based on the post-block sequence reports, the stress group showed lower explicit knowledge of the sequence structure presented in the task, most prominently at the beginning of the task. Thus, stress promoted an aspect of regularity extraction to some extent while disrupted explicit processes.

Considering learning of probability-based regularities, to the best of our knowledge, only one study investigated this aspect of regularity extraction under stress. King [35] found better learning after experimental stress induction on a triplet learning task. In line with this, our study found that stress altered behavioral performance, somewhat boosting the acquisition of probability-based regularities. In detail, it seems that stress induction had an effect only on the extraction of probability-based regularities as group differences only emerged at the beginning of the task, whereas stress did not affect later performance on the task. There are two possible explanations for this effect, which, at this point, cannot be discriminated. First, acquiring probability-based regularities occurs rapidly, often in the early phase of the task, therefore, it is possible that stress boosted the early extraction of probability-based regularities but not the stabilization of the acquired knowledge. The second explanation is related to the change of cortisol levels during the task. Our analysis showed that cortisol levels were comparable immediately after the ASRT task between the groups. As we did not track cortisol levels during the task, we cannot determine in what pace cortisol levels have changed in the stress group. However, speculatively, cortisol levels might have decreased gradually as the task progressed, leading to differences in learning between the stress and control groups only in the early phase of the task. With these information on hand, we cannot take stand on either explanation, i.e., whether stress only boosts the early extraction but not the stabilization of probability-based regularities or stress induction only had a short-term effect, which resulted in group differences only in the early phase of learning. Further studies are needed to directly test and tease apart these possibilities with assessing cortisol levels during the task as well.

As for learning of serial-order regularities, only two prior studies have investigated the acquisition of such regularities under stress. Römer et al. [34] found delayed learning of sequences with deterministic second-order regularities after pharmacological cortisol increase. In detail, both the cortisol and placebo groups showed learning, however, the cortisol group showed decreased learning at first, then similar performance to the placebo group at the end of the task. Dolfen et al. [4] found no effect of stress on learning deterministic sequences after experimental stress exposure. Our results are consistent with the latter study’s result as we also found intact, comparable learning of serial-order information in the stress group after experimental stress induction. As for Römer et al.’s [34] study, it should be taken into consideration that the authors used pharmacological cortisol increase (i.e., oral cortisol), therefore, the results may not be clearly transferred into the stress context. There are substantial differences between administrating stress experimentally and pharmacologically, which influences how stress alters learning as well. Pharmacological manipulation often leads to higher glucocorticoid concentration than a stress induction task [2, 58]. Moreover, stress induction tasks can increase not only the level of cortisol but also trigger the release of many other hormones and neurotransmitters, which can also influence learning [59]. Furthermore, pharmacological manipulation does not result in concurrent noradrenergic activity besides glucocorticoid activity, which occurs, however, after inducing stress experimentally [49]. We can speculate that experimental stress induction does not alter deterministic serial-order learning while pharmacological cortisol increase could decrease it. Since this speculation is based on three studies, further studies are warranted to investigate the effect of stress on the acquisition of serial-order regularities in the context of both pharmacological cortisol increase and experimental stress induction.

Note that the cued version of the ASRT task used in this study [19, 21] enables us to assess the simultaneous extraction of probability-based and serial-order regularities within one learning session. The acquisition of probability-based regularities occurs incidentally (i.e., without awareness or intention to learn) and relatively rapidly [19, 21]. In contrast, the acquisition of the serial-order regularities shows a gradual trajectory and it can be supported by incidental and intentional learning processes as well, with a typically faster acquisition in the intentional learning condition compared to an incidental learning condition [17, 18, 21]. In the cued version of the task, pattern and random elements are visually distinguishable, and participants are instructed to find the repeating sequence of pattern stimuli (i.e., the deterministic serial order in which the pattern stimuli appear throughout the task), which creates a partly intentional learning condition. However, it is important to note that even in an intentional learning situation, RTs tend to reflect a more implicit, incidental measure of the acquired knowledge, while performance on the post-block sequence reports reflects a more explicit knowledge on the deterministic serial order of the pattern stimuli. As typical response times are under 500 ms, the fast pace of the task makes it unlikely that such explicit knowledge would have an impact on participants’ RTs. Therefore, the consciously accessible knowledge of the deterministic serial order of the pattern stimuli (as measured by the post-block sequence report score) and learning of serial-order information (as measured by the RTs) seem to be dissociable measures [57]. The post-block sequence report may be considered as a more explicit measure of the knowledge on the deterministic serial order of pattern stimuli, while the learning RT scores may serve as an implicit measure of serial-order learning, even in an intentional learning condition. In this view, in the present study, stress disrupted the explicit acquisition of the serial-order information, while did not alter the implicit measure of serial-order learning.

Several studies have investigated the effect of stress on learning and memory, including both declarative [e.g., 23, 24] and procedural memory [e.g., 7, 25]. From a broader theoretical perspective, our results can be discussed in the context of the stress-induced memory shift [32, 60]. According to this notion, acute stress can induce a shift from hippocampus-dependent, declarative, cognitively demanding goal-directed learning to striatum-dependent, procedural, habitual forms of learning [32]. One of the first studies demonstrating this shift in humans measured probabilistic classification learning with the Weather Prediction Task [7]. Stressed and control participants showed different learning strategies in the task, with stressed participants preferring procedural strategies and control participants preferring declarative strategies. Neuroimaging results also corroborated these results: in the control condition, task performance correlated positively with hippocampal activity, while in the stress condition, task performance correlated with striatal activity. In this context, speculatively, we could have expected stress boosting both aspects of learning tested in the current study as they are thought to be related to procedural memory and therefore contribute to the acquisition of skills and habits. We also could have expected the disruption of explicit knowledge of the serial order as it may reflect a more declarative, cognitively demanding aspect of learning. Our results are partly consistent with these predictions as the extraction of probability-based regularities was faster and explicit knowledge of the serial order of the pattern stimuli was weaker under stress. Altogether, our results both corroborate and go beyond the notion of stress-induced memory shift by examining procedural memory not as a homogenous system but measuring two aspects related to it. Altogether, further studies are warranted for examining the underlying neural mechanisms to obtain a clearer picture of the effects of stress on the two aspects of learning measured here.

The present study is not without limitations. The effect of stress on the learning of probability-based regularities could be observed only at the beginning of the task and it explained a relatively small variance only. Furthermore, when determining the sample size, we relied on best practices (see Participants section). For reaction time studies with similar designs, Brysbaert and Stevens [61] suggested higher sample size than the one in our study. However, as they explained, having a large number of observations per participant also reduce noise and they suggest that 40 stimuli per condition is good practice for medium effect size. Although we have a lower sample size, our number of observations per participants is much higher: in an epoch, participants were exposed to 400 trials, the whole learning session contained 2000 trials. Another limitation of our study is that we focused only on cortisol response when examining the stress response. As stress affects the body much more expansively, including for example heartbeat and blood pressure, with high individual differences, further studies should examine this more extensively. Hence, we consider these results which suggest that stress boost the extraction of probability-based regularities as exploratory. Future studies are warranted to replicate the findings and further investigate this effect.

In sum, the present study investigated whether the simultaneous acquisition of two types of regularities, namely probability-based and serial-order regularities, is influenced by experimental stress induction. Our results showed that stress affected the acquisition of probability-based regularities as participants in the stress group showed higher learning compared to the control group. Stress did not alter the learning of serial-order regularities but disrupted the explicit knowledge of the repeating sequence, particularly at the beginning of the task. Our results fall in line with previous studies examining the effect of stress on either learning of probability-based or serial-order information [4, 35], however, we went beyond these studies by investigating these learning processes in one experimental design.

## Supporting information

S1 File Supplementary results. Supplementary data analyses on sample with more lenient exclusion criteria and supplementary figures with individual data points.
(DOCX)

S1 Dataset The dataset used for the analyses reported in the manuscript. (XLSX)

S2 Dataset The dataset used for the analyses reported in the S1 File. (XLSX)
