# Supplementary material for: Regularity detection under stress: Faster extraction of probability-based regularities
Source: PLoS One. 2021 Jun 15;16(6):e0253123. doi: 10.1371/journal.pone.0253123 (PMC8205133; doi:10.1371/journal.pone.0253123)
Supplement: S1 File — Supplementary data analyses on sample with more lenient exclusion criteria and supplementary figures with individual data points. (DOCX) [file pone.0253123.s001.docx]

**S1 File**

**Regularity detection under stress: faster extraction of probability-based regularities**

**Running title: Regularity extraction under stress**

Eszter Tóth-Fáber^1,2,3^ Karolina Janacsek^2,3,4 ¶^, Ágnes Szőllősi^3,5^, Szabolcs Kéri^5,6 ¶^, Dezso Nemeth^2,3,7 ¶^ *

* Corresponding author

E-mail: [dezso.nemeth@univ-lyon1.fr](mailto:dezso.nemeth@univ-lyon1.fr) (DN)

^¶^Shared senior authorship

**Supplementary data analyses on sample with more lenient exclusion criteria**

As the number of participants not following task instructions is 10% of all participants, we decided to show the analyses including these seven participants in the Supplementary Material. The analyses are identical to the ones shown in the manuscript. The sample consisted of 60 participants (16 men, 44 women) with a mean age of 20.88 (*SD* = 1.68). The stress group consisted of 31 participants (8 men, 23 women) and the control group consisted of 29 participants (8 men, 21 women). 13 women in the stress group and 8 women in the control group took oral contraceptives regularly.

**Supplementary Results**

**The effectiveness of stress induction**

We used both objective (cortisol levels) and subjective (questionnaire-based ratings) measurements to test the effectiveness of stress induction. For the *objective measurement*, the ANOVA confirmed the effectiveness of the stress induction. We found a significant TIME × STRESS EXPOSURE interaction (*F*(2, 116) = 28.06, *p* < .001, *η^2^_p_ =* .33). The post hoc analysis revealed that cortisol levels in the stress and control groups differed 15 minutes after the stress/control procedure (t2; *M*_stress_ = 32.90, *SD*_stress_ = 15.90, *M*_control_ =21.89, *SD*_control_ = 19.17, *p* = .018), whereas immediately before the stress/control procedure (t1) and immediately after the ASRT task (t3) the two groups’ cortisol levels did not differ significantly (both *p*s > .42, S1 Fig). For the *subjective ratings* of affective state after stress induction or control task, the t-tests showed significantly higher subjective stress, pain and unpleasantness levels in the stress group compared to the control group (see S1 Table).

**S1 Table. Descriptive data of the participants’ subjective stress measurements.**

|  | Group | | | |  |  |
| --- | --- | --- | --- | --- | --- | --- |
|  | Stress (*n* = 31) | | Control (*n* = 29) | |  |  |
|  | *M* | *SD* | *M* | *SD* | *t* | *p* |
| Subjective ratings of stress | 31.19 | 26.28 | 8.51 | 11.98 | -4.24 | <.001 |
| Subjective ratings of unpleasantness | 59.29 | 29.17 | 12.20 | 14.02 | -7.87 | <.001 |
| Subjective ratings of painfulness | 60.00 | 24.89 | 1.20 | 5.61 | -12.42 | <.001 |

*Note.* Immediately after the stress induction or control task, participants rated how stressful, unpleasant and painful (where 0 = not at all, 100 = very) the stress induction or control task was.


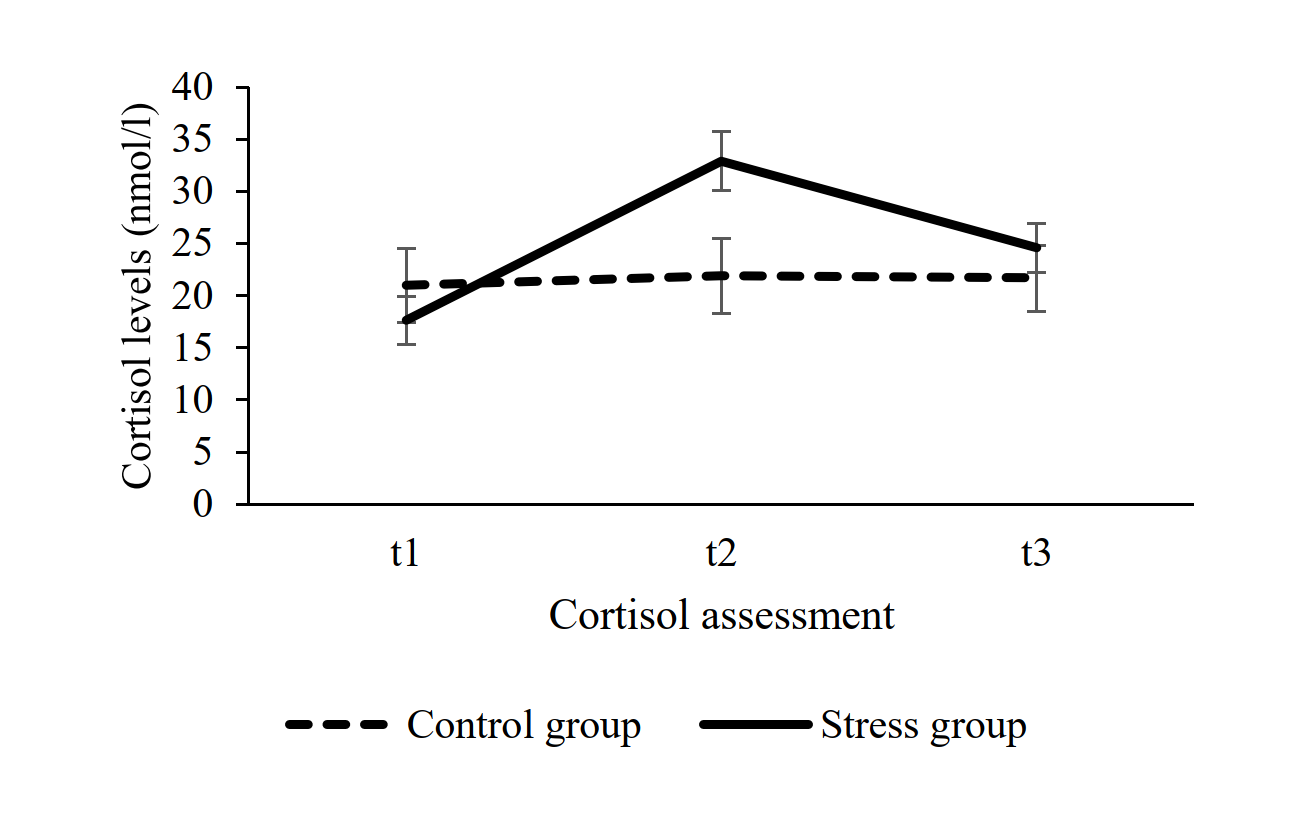


***S1 Fig. The effectiveness of stress induction.*** Salivary cortisol levels were assessed immediately before (t1), 15 minutes after (t2) and 45 minutes after (t3) the stress induction or control task. Error bars denote standard error of mean.

**Average RTs in the practice session**

To compare the two groups’ average speed in the practice session of the ASRT task, a mixed design ANOVA was conducted (see Statistical analysis section in the manuscript). The ANOVA revealed that participants became faster as the task progressed (main effect of BLOCK, *F*(2, 116) = 67.09, *p* < .001, *η^2^_p_ =* .54). Importantly, no group differences were found either in average RTs (main effect of GROUP, *F*(1, 58) = 0.01, *p* = .93) or in the speed-up during the practice blocks (GROUP × BLOCK interaction, *F*(2, 116) = 0.10, *p* = .88).

**The effect of stress induction on the learning of probability-based regularities**

As learning of probability-based regularities is a rapid process [[1](#_ENREF_1),[2](#_ENREF_2)], we focused on the beginning of the task. At first, we examined learning of probability-based regularities only in the first epoch using a mixed-design ANOVA (see Statistical analysis section in the manuscript) between the stress and control groups. Overall, there was no difference in average RTs between the groups (main effect of GROUP, *F*(1, 58) = 1.13, *p* = .29). Participants had faster responses to random high than random low trials (shown by the significant main effect of PROBABILITY, *F*(1, 58) = 31.19, *p* < .001, *η^2^_p_ =* .35), indicating significant learning of probability-based information. Importantly, the stress group showed greater learning of probability-based regularities than the control group (indicated by the significant PROBABILITY × GROUP interaction, *F*(1, 51) = 5.53, *p* = .02, *η^2^_p_ =* .09, stress group: *M_random high_* = 378.48 ms, *M_random low_* = 396.55 ms, learning score: *M* = 18.07 ms; control group: *M_random high_* = 398.28 ms, *M_random low_* = 405.64 ms, learning score: *M* = 7.36 ms).

To explore the trajectory of learning of probability-based information in the stress and control groups, we conducted a mixed-design ANOVA on the RTs (see Statistical analysis section in the manuscript). There were no differences in average RTs between the groups (main effect of GROUP, *F*(1, 58) = 1.74, *p* = .19). Participants became faster with practice on both trials (indicated by the significant main effect of EPOCH, *F*(4, 232) = 6.07, *p* = .01, *η^2^_p_ =* .095). Over groups, RTs were faster on random high trials than on random low trials (shown by the significant main effect of PROBABILITY, *F*(1, 58) = 155.70, *p* < .001, *η^2^_p_ =* .73), indicating significant learning of probability-based regularities. The RT differences between random high and random low trials increased with practice (indicated by the significant PROBABILITY × EPOCH interaction, *F*(4, 232) = 2.85, *p* = .03, *η^2^_p_ =* .05). Importantly, the time course of learning was different between the groups (indicated by the marginally significant GROUP × PROBABILITY × EPOCH interaction, *F*(4, 232) = 2.08, *p* = .08, *η^2^_p_ =* .04, see S2 Fig). Follow-up analysis on the learning scores revealed a significant difference in the first epoch: the stress group showed greater learning of probability-based regularities than the control group (stress: *M* = 18.07 ms, control: *M* = 7.36 ms, *p* = .02). Additionally, the stress group showed marginally greater learning of probability-based regularities in the third epoch as well (stress: *M* = 24.11 ms, control: *M* = 15.03 ms, *p* = .08), while there were no group differences in the remaining epochs (all *p*s > .22). Other interactions did not reach significance (all *p*s > .17).


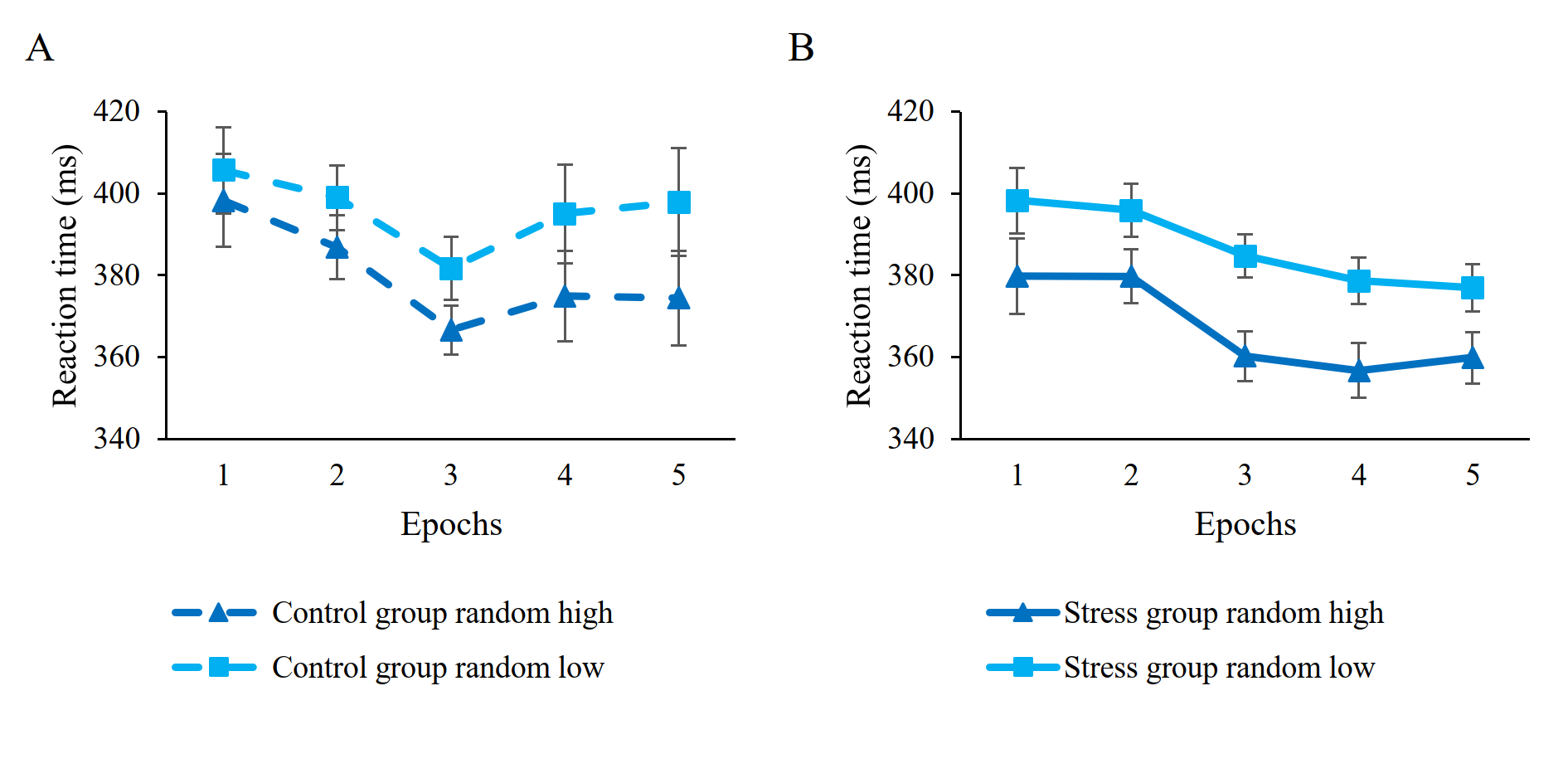


***S2 Fig. Learning of probability-based regularities in (A) control and (B) the stress groups.*** Dashed lines represent the control group, continuous lines represent the stress group. Darker blue lines with triangle symbols indicate reaction times on the random high trials, light blue lines with square symbols indicate reaction times on the random low trials. Learning of probability-based regularities is quantified by the distance between dashed and continuous lines, greater distance represents better learning. Error bars denote standard error of mean.

**The effect of stress induction on the learning of serial-order regularities**

As learning of serial-order regularities is a gradual process [[1](#_ENREF_1),[2](#_ENREF_2)], at first, we investigated learning between the groups in the last epoch using a mixed-design ANOVA (see Statistical analysis section in the manuscript). Overall, participants showed similar average reaction times (main effect of GROUP, *F*(1, 58) = 0.50, *p* = .48). Participants showed faster responses to pattern than to random high trials (main effect of ORDER, *F*(1, 58) = 9.41, *p* = .003, *η^2^_p_ =* .14), indicating significant learning of serial-order regularities. The analysis showed comparable learning between the groups (shown by the non-significant ORDER × GROUP interaction, *F*(1, 58) = 0.58, *p* = .45).

To examine the trajectory of learning of serial-order regularities on the whole task, we also used mixed-design ANOVA on the RT (see Statistical analysis section in the manuscript). Average RTs did not differ between the groups (main effect of GROUP, *F*(1, 58) = 1.24, *p* =.27). With practice, participants showed faster RTs on both trials (shown by the significant main effect of EPOCH, *F*(4, 232) = 41.61, *p* < .001, *η^2^_p_ =* .42). Participants showed faster RTs on pattern trials compared to random high trials (indicated by the main effect of ORDER, *F*(1, 58) = 14.95, *p* < .001, *η^2^_p_ =* .21), indicating significant learning of serial-order regularities. Moreover, the RT differences between pattern and random high trials gradually increased with practice (shown by the significant ORDER × EPOCH interaction, *F*(4, 232) = 4.05, *p* = .04, *η^2^_p_ =* .07, see S3 Fig). Other interactions did not reach significance (all *p*s > .47).


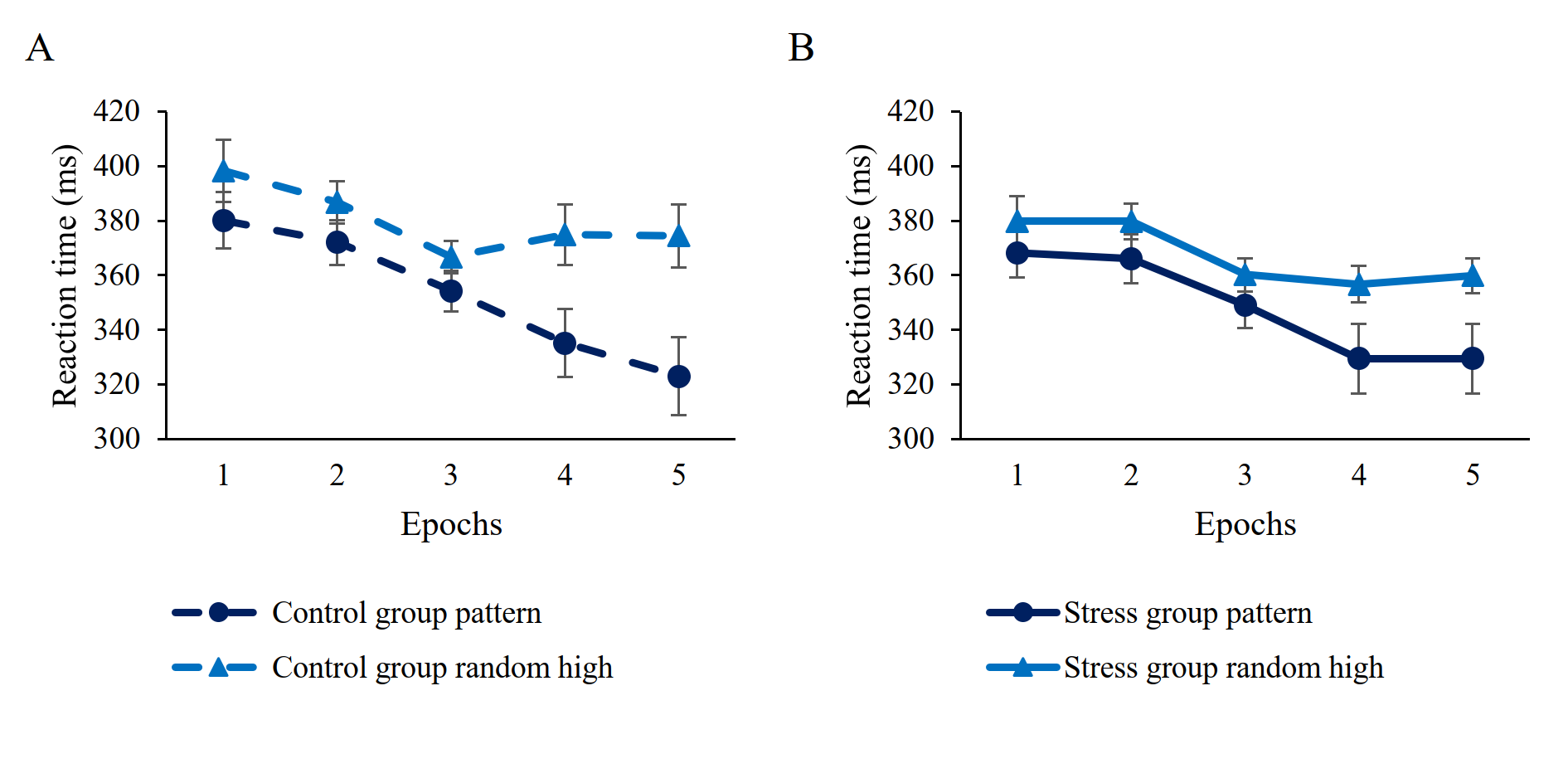


***S3 Fig. Learning of serial-order regularities in (A) control and (B) the stress groups.*** Dashed lines represent the control group, continuous lines represent the stress group. Dark blue lines with circle symbols indicate reaction times on pattern trials, lighter blue lines with triangle symbols indicate reaction times on random high trials. Learning of serial-order regularities is quantified by the distance between dashed and continuous lines, greater distance represents better learning. Error bars denote standard error of mean.

Performance on *post-block* *sequence reports,* reflecting explicit sequence knowledge, was also compared between the groups. To investigate the change in explicit sequence knowledge during the task, we conducted a mixed-design ANOVA with GROUP (stress vs. control) as between-subjects factor and EPOCH (1-5) as within-subject factor. Averaged over the epochs, the stress group showed similar explicit sequence knowledge than the control group (main effect of GROUP, *F*(1, 58) = 0.41, *p* = .52, *M*_stress_ = 86.2%, *M*_control_ = 89.4%). Explicit sequence knowledge increased as the task progressed in both groups (*F*(4, 232) = 9.43, *p* < .001, *η^2^_p_ =* .14), however, the time course of this increase differed in the groups (indicated by the significant GROUP × EPOCH interaction, *F*(4, 232) = 4.82, *p* = .005, *η^2^_p_ =* .08). Pairwise comparisons showed that the control group had comparable explicit sequence knowledge in all epochs, which varied between 86.8% and 90.9% (all *p*s < .16). In contrast, the stress group showed significantly lower explicit sequence knowledge in the first epoch (*M* = 74.6%) compared to the remaining epochs (*M* > 84.7%, all *p*s < .001). Then, they showed mostly comparable explicit sequence knowledge in Epoch 2-5, which varied between 87.4% and 93.5% (all *p*s > .065, except for the pairwise comparison between Epoch 2 and Epoch 5, where Epoch 2 < Epoch 5, *p* = .001 and between Epoch 4 and Epoch 5, where Epoch 4 < Epoch 5, p = .016).

Supplementary Figures with individual data points

**
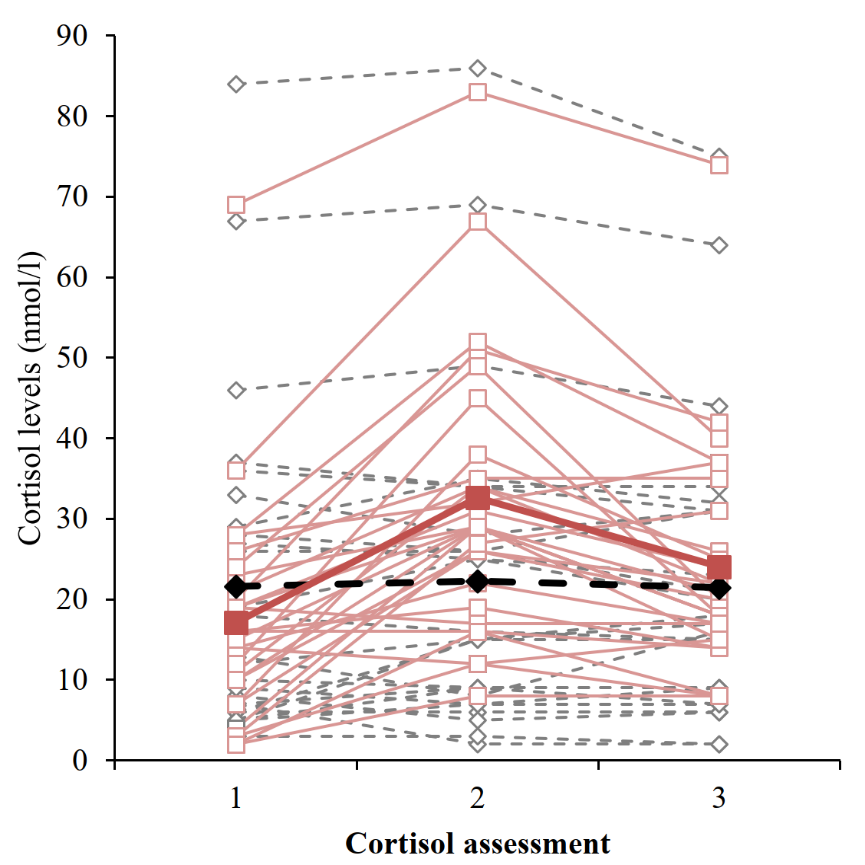
**

***S4 Fig. The effectiveness of stress induction with individual data points.*** Salivary cortisol levels were assessed immediately before (t1), 15 minutes after (t2), and 45 minutes after (t3) the stress induction or control task. Dashed gray lines with diamond symbols represent the control group, continuous red lines with square symbols represent the stress group. Each light gray and light red line represent a participant’s cortisol levels, while the groups’ average cortisol levels are presented in black color for the control group and darker red color for the stress group.


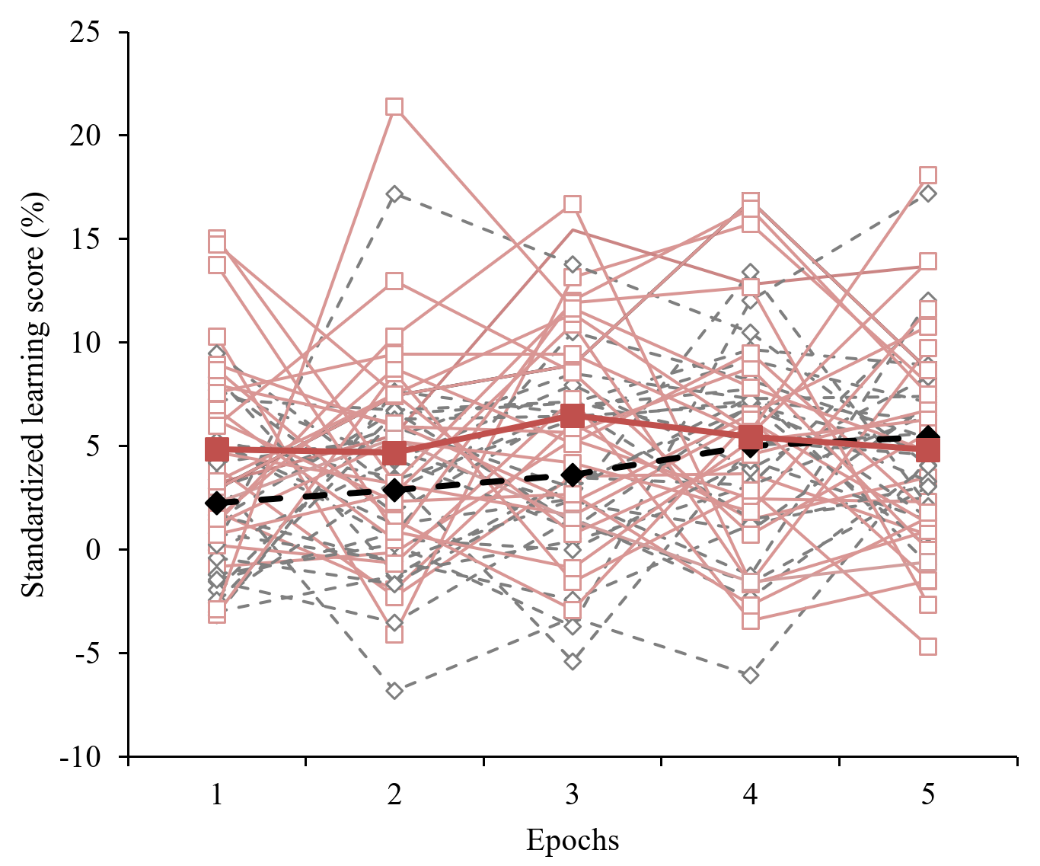


***S5 Fig.*** ***Learning of probability-based regularities in the control and stress groups with individual data points.*** Dashed gray lines with diamond symbols represent the control group, continuous red lines with square symbols represent the stress group. Here, instead of reaction times for random high and random low trial types, the learning scores (i.e., the difference in reaction times between random high and random low trial types) are presented to help interpretability. Higher learning score indicates better learning. Each light gray line and light red line represent a participant’s learning score, while the groups’ average learning scores are presented in black color for the control group and darker red color for the stress group.


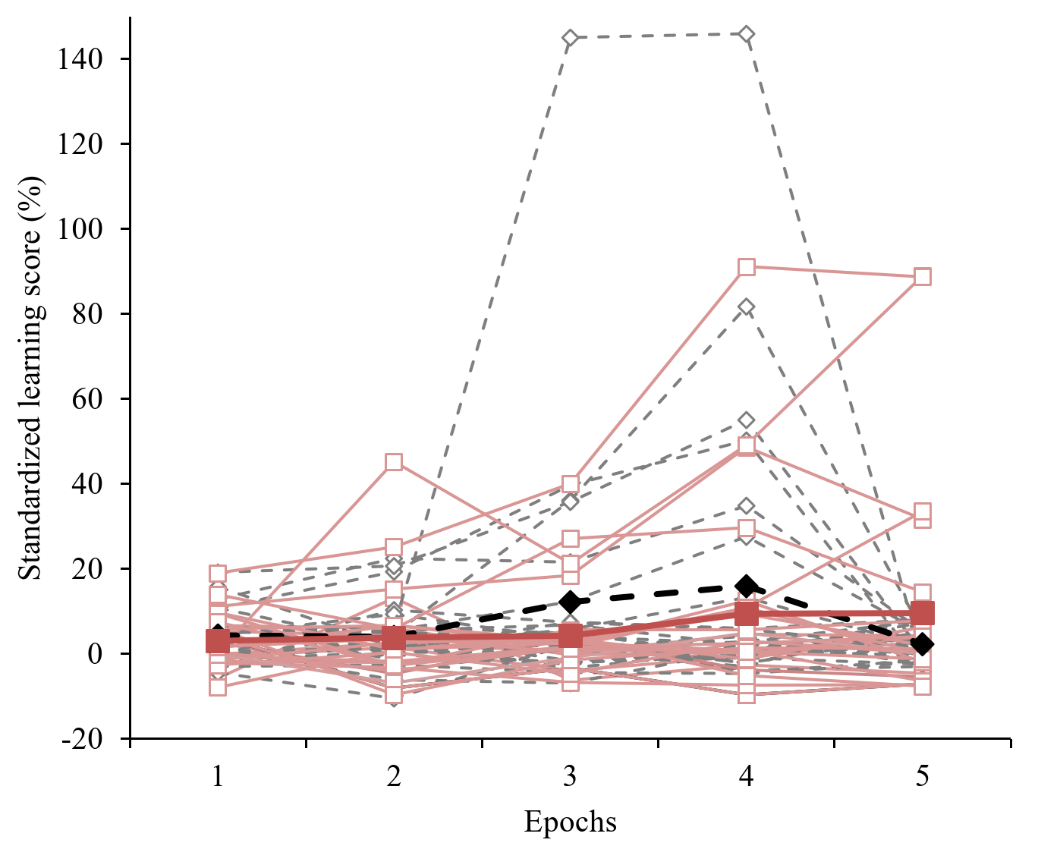


***S6 Fig.*** ***Learning of serial-order regularities in the control and stress groups with individual data points.*** Dashed gray lines with diamond symbols represent the control group, continuous red lines with square symbols represent the stress group. Here, instead of reaction times for pattern and random high trial types, the learning scores (i.e., the difference in reaction times between pattern and random high trial types) are presented to help interpretability. Higher learning score indicates better learning. Each light gray line and light red line represent a participant’s learning score, while the groups’ average learning scores are presented in black color for the control group and darker red color for the stress group.

**References**

1. Kóbor A, Takács Á, Kardos Z, Janacsek K, Horváth K, et al. (2018) ERPs differentiate the sensitivity to statistical probabilities and the learning of sequential structures during procedural learning. Biological psychology 135: 180-193.

2. Simor P, Zavecz Z, Horvath K, Elteto N, Török C, et al. (2019) Deconstructing procedural memory: Different learning trajectories and consolidation of sequence and statistical learning. Frontiers in Psychology 9: 2708.
